# In-hospital interventions to promote relational practice with families in acute care settings: A scoping review

**DOI:** 10.4102/hsag.v27i0.1694

**Published:** 2022-02-14

**Authors:** Waheedha Emmamally, Christen Erlingsson, Petra Brysiewicz

**Affiliations:** 1 Discipline of Nursing, School of Nursing and Public Health, University of KwaZulu-Natal, Durban, South Africa; 2 Department of Health and Caring Sciences, Faculty of Health and Life Sciences, Linnaeus University, Kalmar, Sweden

**Keywords:** acute care setting, collaborations, family engagement, family-healthcare professional interactions, relational practice

## Abstract

Relational practice is characterised by genuine interaction between families and healthcare professionals that promotes trust and empowerment. Positive clinical outcomes have been associated with relational practice. To assess and examine in-hospital interventions designed to promote relational practice with families in acute care settings of emergency departments, intensive care units and high care units. The preferred reporting Items for Systematic Reviews and Meta-Analyses guidelines informed the design of this scoping review. To identify relevant studies, databases (Academic Search Complete; CINAHL; PubMed; PsyInfo) and the search engine Google Scholar were searched using terms for core elements of relational practice and family engagement. Of the 117 articles retrieved, eight interventional studies met the search criteria. The interventions focused on relational practice elements of collaborating with and creating safe environments for families, whilst only one addressed healthcare professionals being respectful of families’ needs and differences. In relation to the nature of engagement of families in interventions, the focus was mainly on improving family functioning. Family engagement in the interventions was focused on involving families in decision-making. The scoping review revealed a limited number of in-hospital interventions designed to promote relational practice with families in acute care settings. Further research is encouraged to develop such interventions.

**Contribution:**

The scoping review has highlighted specific elements of relational practice that have been overlooked in the mapped interventions. This provides guidance on where future interventional research may be focused.

## Introduction

Families play an important role in caring for their loved ones in acute healthcare settings, whilst simultaneously assisting healthcare professionals (HCPs) with vital information for the treatment of the patient (Bhalla et al. 2014). Moreover, research has demonstrated that HCP and family collaboration in acute care settings leads to positive patient outcomes of recovery and satisfaction with care (Indovina et al. 2016; Williams, Nolan & Keady 2009). Inclusive of positive relationships between HCPs and families is the relational practice approach, which is defined as an approach that invests in creating meaningful relationships between individuals (Zou 2016). Relational practice with families in healthcare settings centres on HCPs and family members who enter into a relationship, fully prepared to share their true personalities and grow together (Jordan 2010). An important aspect of collaborating with families is to know how families define themselves, as this definition directs the role and expectations of families during the illness experience of their loved ones. For this reason, the current study adopted Doane and Varcoe’s (2007) definition of a family, as a relational process where family members are interlinked with their experiences, emotions and social circumstances.

Core elements of relational practice include individuals consciously connecting and growing towards each other, authenticity in caring, whereby individuals are transparent and genuine in their emotions, being attuned to each other’s needs whilst honouring differences, mutual trust and respect between individuals leading to self-empowerment (Fletcher 1998; Jordan 2010). Self-reflection in relational practice encourages HCPs to confront prejudices that may be present in family encounters (Duffey & Somody 2011; Hartrick 2008). Relational practice is about HCPs creating safe environments for families through therapeutic communication (Doane & Varcoe 2007). The authors elaborate that in creating safe environments, HCPs promote feelings of security that facilitates families to share their emotions. Healthcare professionals are encouraged to acknowledge the contextual factors that may shape a patient’s and family’s responses to experiences and interactions with people (Zou 2016). These include personal characteristics, and socio-political, cultural and geological factors that affect how patients and families manage their illness. Jordan (2010) speaks about the element of HCPs being fully involved in relationships with families thus supporting families to grow.

Equally important to a relational practice approach is the active engagement of families in their care (Shields 2015). Family engagement requires that HCPs actively partner with families, acknowledging that families hold the expertise to improve their healthcare experiences (Burns et al. 2018). Family engagement speaks to HCPs working with families at every level of the healthcare system to transform care whilst preserving their dignity (Shield 2015). *Three dimensions are proposed when reviewing* family engagement in an intervention, namely, the focus of the intervention, the structure of the family engagement and the level of family engagement (Knafl et al. 2017). Knafl et al. (2017) explains that when a family is actively engaged in an intervention, the intervention becomes relevant and acceptable to them. Workload pressures, a rapid-paced environment and high patient acuities in the acute care settings often challenge relational interactions and active engagement with families (Hetland et al. 2018). Amidst these challenges, HCPs working in acute care settings need guidance to meaningfully connect with families (Östlund & Persson 2014). It is therefore important to identify whether existing interventions which are designed to promote family and HCP collaboration address the core elements of relational practice and the nature of family engagement. To this end, the review aims to assess and examine in-hospital interventions designed to promote relational practice with families in acute care settings of emergency departments (EDs), intensive care units (ICUs) and high care units (HCUs).

## Methods

### Research design

A scoping review following the preferred reporting items for Systematic Reviews and Meta-Analyses (Page et al. 2021) and the five-stage framework proposed by Arksey and O’Malley (2005) was conducted. A scoping review methodology was chosen over other reviews as it allowed for a broad overview of key concepts on the relatively understudied area of relational practice (Colquhoun et al. 2014).

#### Stage 1: Identifying the research questions

The authors formulated the research questions guided by the population, concept and context (PCC) and closely aligned to the aim of the review. The research questions were as follows: 1) What in-hospital interventions are available to promote relational practice with families in acute care settings of EDs, ICUs and HCUs? 2) What elements of relational practice did the interventions address? 3) What was the nature of family engagement in the interventions?

#### Stage 2: Identifying relevant studies

A search strategy detailing search terms (see Table 1) and identified databases were developed in consultation with a specialist librarian. Search terms related to relational practice and family engagement were applied to the following databases: Academic Search Complete, CINAHL, MEDLINE and PubMed, PsyINFO and the search engine Google Scholar. After selecting relevant titles from the initial results, the authors identified additional keywords to refine the search. The inclusion criteria of the review included: A population of HCPs working in acute care settings and family members visiting acute care settings; the concept was in-hospital interventions occurring in the acute care settings (namely EDs, ICUs and HCUs) and the context of the studies were the nature of family engagement and elements of relational practice. Studies had to include outcome measures related to a specific core element of relational practice with family members. The core elements are included in Table 3.

**TABLE 1:** Search terms used in electronic databases.

Context	Concept	Population
Collaborative relationship with families	Emergency service	Acute care doctors
Engagement with families	Emergency room	Acute care physicians
Consciously relating to families	Emergency department	High care nurses
Partnering with families	Emergency units/centre	Acute care nurses
Empowering families	Accident and emergency	Acute care clinicians
Therapeutic relationship with families	Trauma outpatients	Emergency care nurses
Connecting with families	Casualty department	Trauma nurses/doctors
Genuinely interacting with families	Emergency setting	Acute care nursing personnel
Mutuality in relationships with families	Trauma unit Adult intensive care units	Emergency department staff Accident and emergency staff
Growth-fostering relationships with families	Adult critical care units High care units Emergency department	Emergency physicians Critical care nurses Intensivists Intensive care nurses Acute healthcare professionals

#### Stage 3: Study selection

**FIGURE 1:**
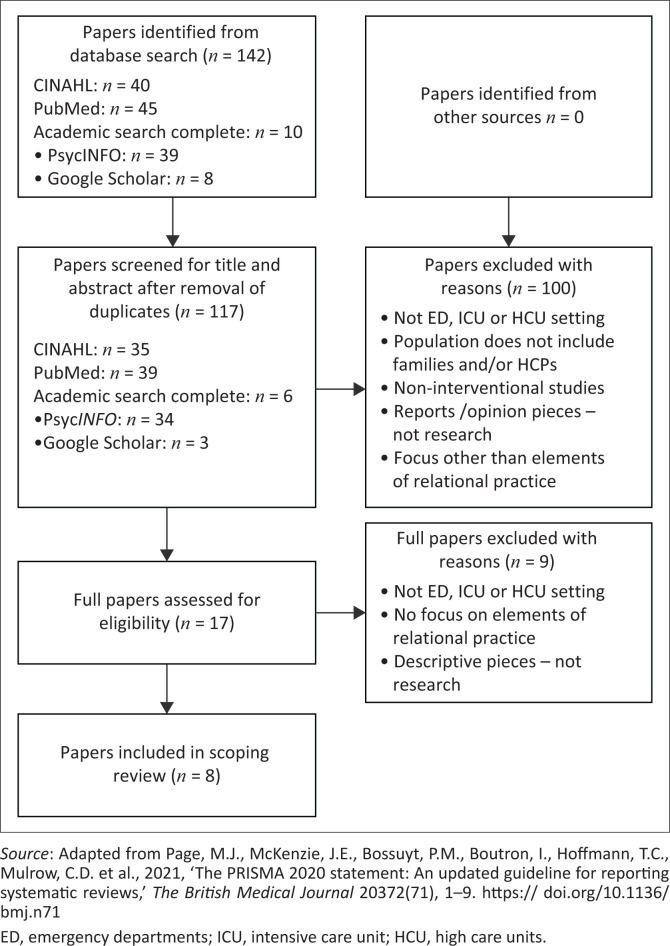
PRISMA flow diagram of the review process.

Three authors were involved in the review process. Articles in English which were published between January 2005 and December 2018 were included in the review and this was informed by the interest in relational practice and quality outcomes of complex healthcare contexts in the literature (Williams et al. 2009). The database search yielded 142 papers with 25 duplicate papers being removed by the first author (WE). The remaining 117 papers underwent a two-phase review process involving two authors (WE and PB) working independently. Two authors screened the titles and abstracts against the inclusion criteria and a further 100 papers were excluded. The remaining 17 papers underwent a full-text assessment by the same independent authors. This resulted in eight papers for the final review. After each stage, the two authors met to discuss the results of their independent review and resolve any emerging issues, with discrepancies being resolved by the third author (CE). Figure 1 shows the review process.

#### Stage 4: Charting the data

A data extraction table was developed by the reviewers for relevant data extraction and management of the eight papers included in the final review.

#### Stage 5: Collating, summarising and reporting the results

The three research questions of the scoping review guided this stage of collating, summarising and synthesising the studies. The details of the included studies are shown in Table 2.

**TABLE 2:** Articles included in review (*n* = 8).

Citation	Location	Aim	Design	Setting	Participants	Tools used	Intervention	Intervention outcomes	Recommendations and conclusions
White et al. (2018)	USA	Compare a multi-component family-support intervention with usual care that is given.	Stepped-wedge cluster randomised trial	1 neuro-surgical ICU, 1 transplant ICU, 2 medical-surgical ICUs, and 1 medical ICU	429 family members (surrogate decision-makers) in intervention group and 677 in control group	Symptoms assessed using Hospital Anxiety and Depression Scale	Comprised of 3 components: 1) Nurses trained on interpersonal skills 2) Family support pathway and 3) Management support for implementation of intervention.	1. No significant differences in the surrogates’ symptoms of anxiety & depression, between the intervention and control group, six months post implementation of the intervention. 2. ↑ in quality of decision-making and clinician family communication.	It is feasible to train an interprofessional ICU team to provide a family support intervention.
Jacobowski et al. (2010)	USA	Pilot investigation to explore the effects of early, consistent communication with families, by adding a family component to interdisciplinary ICU rounds.	Before/after study of a pilot project	26 bedded medical ICU	227 family members were interviewed	Family Satisfaction in ICU	Family rounds consisting of two family members joining in the interdisciplinary round. 1) The physician gave summarised reports to family members and families were encouraged to participate. 2) Clinician–family meetings could be arranged on family’s request.	Participation in family rounds was associated with family satisfaction on the frequency of physician communication and support during decision-making.	More work is needed to optimise communication between ICU personnel, patients and families.
Blackwell et al. (2017)	UK	To critique the feasibility of an experience-based Co-design as a quality improvement intervention in complex healthcare settings.	Experience-based co-design	ED within teaching hospital	ED staff, palliative care team, patients in ED, family caregivers	Semi-structured interviews with staff Narrative interviews with families	A co-design event involving staff, patients and family members, Creation of a DVD of patient–family–staff experiences for reflective discussions.	The study identified quality improvement initiatives to enhance emergency department palliative care processes.	To test alternative ways of increasing patients’/ families’ input during the co-design phase.
Svavarsdottir et al. (2015)	Iceland	To report on approaches that were used to assist with implementation of family systems nursing (FSN) at a university hospital.	1st phase Quasi experimental design 2nd phase Cross-sectional research design	1st phase – all divisions of the hospital except the ED 2nd phase – all divisions of the hospital	1st phase – 457 nurses 2nd phase – 812 nurses	The Family Importance in Nursing Care Nurses’ Attitude Questionnaire The Nurses Knowledge and Confidence Scale in Using Family Systems Nursing In Clinical Practice	Education and training programme to equip nurses in assessing families and offering appropriate emotional and educational intervention based on the Calgary models.	Nurses who participated in a course on FSN and the ETI programme indicated a readiness for applying FSN to practise.	To continue to support nurses who have taken the programme, and to offer the programme to nurse who still have not taken it.
Mitchell et al. (2009)	Australia	Evaluate effects of family-centred care of critical care nurses partnering with patients’ families in providing basic care.	Pragmatic clinical trial with a non-equivalent control group, pre-test–post-test design	Two setting, both combined surgical and medical ICUs	174 family members of critical care patients	Family-centred Care Survey	ICU nurses identified care options to involve families. At the intervention site family members participated with ICU nurses in providing basic care to their loved one.	A significant ↑ in in respect, collaboration and support scores of families was evident 48 h post intervention.	1) Data on qualitative components of families’ and nurses’ experiences of family support interventions are needed. 2) Development of more formalised approaches to include families in care and meet their needs is advocated.
Chien et al. (2006)	Hong Kong	To examine the effects of a needs-based education programme provided within the first three days of patients’ hospitalisation, on the anxiety levels and satisfaction of their families.	Quasi experimental study - Pre- and post-test design	One ICU in Hong Kong	66 family carers	Critical Care Family Needs Inventory	Needs-based education programme consisting of: 1) 1-hour educational session on consecutive days for each family carer participant based on needs assessment from the pre-test. 2) Provision of a pamphlet with important information on ICU. 3) Nurse-initiated interaction with families. 4) Daily follow-up family telephone calls to answer family questions and allay anxieties.	Post intervention the experimental group reported ↓ levels of anxiety and ↑ levels of satisfaction compared to the control group.	Formulations of a family education programme should be based on a family needs assessment.
Van Mol et al. (2016)	Netherlands	To evaluate the impact of supportive interventions perceived by both the intensive care unit patients’ relatives and the healthcare providers.	Time trend quantitative design	Four different ICUs in a large university medical centre	Year 2012 – 211 family members Year 2013 – 123 family members	Consumer Quality Index ’Relatives in ICU’	Multi-interventional programme comprising: 1) Intake interview with families to provide information and encourage discussions of emotions. 2) Motivating families to keep a journal of their reactions and emotions. 3) Weekly psychosocial round with a social worker, intensivist and spiritual counsellor.	Family perception regarding quality care especially on giving of information	1) Staff must be trained to meet the psychosocial needs of families. 2) Efforts to change mind-sets of professionals to families is important to improve quality care in ICU.
Eggenberger and Sanders (2016)	USA	To examine the influence of an educational intervention on nurses’ attitudes and confidence in providing family care. To examine families’ perceptions of support from nurses in an adult critical care setting.	Pre- and post mixed methods design	ICU	Nurses in ICU and family members	Pre-intervention tool with families: Iceland Family Perceived Support Questionnaire Pre and post intervention tools with nurses: Focus group and Family Nurse Practice Scale	Educational intervention for nurses was a 4-hour workshop. 1) Content included understanding nurse and the families’ experience of the illness, strategies of therapeutic conversations, digital stories and role playing. 2) Nurses received reference manual of research focused on families’ illness experiences & family interventions.	Nurses reported ↑ confidence, knowledge, and skills following the intervention.	More research needed to understand the impact of an educational intervention in clinical practice to encourage change in nurses’ perception and knowledge on families. Nurses understanding of a family’s experience is the first step to influence practice.

ICU, intensive care unit

## Review findings

### Overview of interventions in included studies

All eight studies originated in developed countries. Three studies originated in the United States and the remaining five originated in the United Kingdom, Iceland, the Netherlands, Hong Kong and Australia (Table 2). Most of the studies were conducted in an ICU setting (Chien et al. 2006; Eggenberger & Sanders 2016; Jacobowski et al. 2010; Mitchell et al. 2009; Van Mol et al. 2016; White et al. 2018), one in the ED (Blackwell et al. 2017), and one study was conducted across hospital departments, including the ICU setting (Svavarsdottir et al. 2015). The studies were primarily quantitative in approach.

With regard to the participants who were targeted by the intervention, five studies focused on nurses and family members (Chien et al. 2006; Eggenberger & Sanders 2016; Mitchell et al. 2009; Svavarsdottir et al. 2015; White et al. 2018). The interventions of the remaining three studies targeted multidisciplinary team participation, namely those of Jacobowski et al. (2010) (medical doctors, families, nurses); Van Mol et al. (2016) (families, nurses, spiritual care workers, a social worker, a psychologist, medical doctors – intensivists) and Blackwell et al. (2017) (patients, family members, a medical doctor-palliative care, nurses and ED management staff).

Seven studies used multicomponent family interventions. These were educational and/or psychological support programmes for families (Blackwell et al. 2017; Chien et al. 2006; Jacobowski et al. 2010; Van Mol et al. 2016), educational and training programmes for nurses (Eggenberger & Sanders 2016; Svavarsdottir et al. 2015), and a support programme for families with an educational component for nurses (White et al. 2018). The study by Mitchell et al. (2009) described a single component intervention of involving family members in the basic care of their loved one admitted in ICU. All studies described the component/components of the interventions in detail.

Seven studies conducted pre- and post-test measurements using validated quantitative tools (Chien et al. 2006; Eggenberger & Sanders 2016; Jacobowski et al. 2010; Mitchell et al. 2009; Svavarsdottir et al. 2015; Van Mol et al. 2016; White et al. 2018). Majority (*n* = 5) of the studies reported that the interventions had positive outcomes of improved family support and improved family decision-making (Chien et al. 2006; Jacobowski et al. 2010; Mitchell et al. 2009; Van Mol et al. 2016; White et al. 2018). Outcomes of increased clinician skills and confidence were noted in the interventions of Svavarsdottir et al. (2015); Eggenberger and Sanders (2016); Blackwell et al. (2017) and White et al. (2018). Regarding reports of sustainability of the interventions, three studies discussed plans to ensure sustainability of the interventions (Blackwell et al. 2017; Svavarsdottir et al. 2015; Van Mol et al. 2016). The contents of the interventions are detailed in Table 2.

### Elements of relational practice addressed by the interventions

The authors utilised the core elements of relational practice described by Duffey and Somody (2011); Fletcher (1998); Hartrick (2008), Jordan (2010); Doane and Varcoe (2007) and Zou (2016) to answer the question of ‘What elements of relational practice did the interventions address?’ (Refer to Table 3). All eight studies focused on relational elements of, *consciously preparing to collaborate and involve families through authentic connection* and *creating safe environments through actions of therapeutic communication & providing information.* Comparatively, only two studies (Eggenberger & Sanders 2016; Svavarsdottir et al. 2015) addressed relational elements of *empathetic understanding of families through self-reflection by the HCPs.* The relational element of *appreciating the factors that influenced experiences and relationships* was evidenced in the intervention by Blackwell et al. (2017) and Chien et al. (2006). Finally, Chien et al. (2006) *looked at the relational practice element of HCPs being attuned to families’ needs whilst honouring their cultural and social differences*.

**TABLE 3:** Elements of relational practice (*n* = 8).

Elements of relational practice	Chien et al. (2006)	Mitchell et al. (2009)	Jacobowski et al. (2010)	Svavarsdottir et al. (2015)	Eggenberger and Sanders (2016)	Van Mol. et al. (2016)	Blackwell et al. (2017)	White et al. (2018)
*Consciously preparing to collaborate and involve families through authentic connection* (Fletcher 1998; Jordan 2010)	x	x	x	x	x	x	x	x
*Creating safe environments through actions of therapeutic communication and providing information* (Doane & Varcoe 2007)	x	x	x	x	x	x	x	x
*Creating safe environments through therapeutic actions of sharing emotional impact of illness* (Doane & Varcoe 2007)	x				x	x	x	x
*Being fully present in a relationship, sharing goals and fostering growth of all individuals through involvement of families* (Fletcher 1998; Jordan 2010)	x	x	x			x	x	x
*Being fully present in a relationship, sharing goals and fostering growth of all individuals Increasing family capacity for decision making* (Fletcher 1998; Jordan 2010)			x			x	x	x
*Mutual respect, empathy and trust that leads to mutual empowerment* (Brown 2016)	x	x					x	
*Self-reflection to challenge status quo leads to development of relational capacity of HCP* (Duffey & Somody 2011)				x	x	x	x	x
*Self-reflection leads to empathetic understanding* (Duffey & Somody 2011)				x	x			
*Appreciating the inter, intra and contextual factors that influence experiences and relationships* (Zou 2016)	x						x	
*Being attuned to families’ needs whilst honouring differences* (Fletcher 1998; Jordan 2010)	x							

HCPs, healthcare professionals.

### Nature of family engagement in the interventions

The nature of family engagement in the interventions of the reviewed studies was analysed using the three dimensions of family engagement in an intervention, namely, the focus of the intervention, the structure of the family engagement and the level of family engagement (Knafl et al. 2017). Regarding the focus of the intervention, four of the studies focused on improving family relationships to optimise family functioning (Eggenberger & Sanders 2016; Jacobowski et al. 2010; Svavarsdottir et al. 2015; Van Mol et al. 2016). The structure of family engagement involved key family figures in the intervention (Blackwell et al. 2017; Chien et al. 2006; Jacobowski et al. 2010; Mitchell et al. 2009; Van Mol et al. 2016; White et al. 2018). Family engagement in the interventions of the studies was the active involvement of families in decision-making (Eggenberger & Sanders 2016; Jacobowski et al. 2010; Mitchell et al. 2009; Svavarsdottir et al. 2015; Van Mol et al. 2016; White et al. 2018).

## Discussion

A limited number of studies *(n = 8)* were retrieved that included interventions for promoting relational practice with families in acute care settings. All the studies in the review were conducted in developed countries, where health resources, cultural and social perspectives of a family’s role during illness and hospitalisation and the family’s experience of illness may be different from that of developing countries (Shields 2015). According to the author, these differences play a crucial role in determining whether interventions maybe effectively translated to socially and culturally diverse populations. It is accordingly suggested that future studies be carried out in developing countries to provide valuable information on the socio-cultural and economic factors that may affect the development of family focused interventions.

The reviewed studies used different study designs with two studies being randomised controlled trials. Vincent (2010) stated that conducting randomised controlled trials in complex healthcare settings may be limited because of problems with timing and working with culturally diverse populations, adding that it may also be unrealistic to conduct studies on relationships in controlled environments. All the studies in the review used validated quantitative measures to assess the impact of the intervention on the participants. However, a limitation in using primarily quantitative measures to collect data is that quantitative measures do not capture the unique emotions and experiences of participants (Edelstein et al. 2017). It is recommended that future studies include qualitative approaches that are advantageous in collecting rich data on participant experiences, as well as mixed methods approaches, where quantitative and qualitative study approaches complement each other.

Similar to other interventional studies by Torke et al. (2016) and Heyland et al. (2018), the majority of the reviewed studies described multiple-component interventions. However, there is no compelling evidence that indicates whether multicomponent interventions are more effectively translated to practice than single-component interventions (Eldh & Wallin 2015; Squires et al. 2014). The majority of the reviewed studies focused on nurses as participants in their interventions. This may be attributed to the idea that collaboration and support of families are viewed as a nursing responsibility (Malliarou et al. 2014), or that nurses have a unique and constant relationship with families and patients and are thus best suited for interventions with families (Adams et al. 2014). However, collaborating with families must be a shared goal of all HCPs (Casimiro et al. 2015) and to this end, future interventions should strive to include all HCPs in family focused interventions.

When considering the outcomes of the interventions reported in the included studies, the family members indicated that their perceived expectations and needs were met by the interventions. Torke et al. (2016) recounted similar results in their studies, concluding that family and HCP collaboration may be improved with family members being involved in intervention development. Only a few reviewed studies included plans to sustain the interventions that had been developed. The importance of reporting on plans for sustainability is that it allows for maximisation of resources, realisation of health outcomes and on-going support and engagement by participants (Walugembe et al. 2019).

It was notable, that the interventions in the review, developed for acute care settings did address some elements of relational practice. Previous research has called for strategies to support family collaboration in acute care setting (Mackie, Marshall & Mitchell 2017).The element of *HCPs respecting families’ needs and honouring family differences in terms of their values systems and practices* (Fletcher 1998; Jordan 2010) were addressed in only one of the reviewed studies. This finding maybe attributed to the fact that the studies were conducted in acute care settings, which are known to as task-oriented healthcare settings (Lloyd, Elkins & Innes 2018). The workload and time pressures often restrict HCP’s interactions with families.

The nature of family engagement in the interventions varied, according to the dimensions proposed by Knafl et al. (2017). Although most studies focused on optimising family functioning through the interventions, the interventions concentrated only on key family members. This may reflect limited attention to considering the family as a unit of care, that is, where each family member contributes to the well-being of the other and the family context impacts on the success of family-focused interventions (Knafl et al. 2017). The interventions of the current review involved family members as active participants. This can be contrasted to the results of a scoping review by Goodridge et al. (2018), which revealed that the family engagement in interventions was confined to family members receiving information. Engaging actively with family members indicates a possible movement of HCPs towards acknowledgement that families possess the expertise to contribute their own healthcare by virtue of their unique life experiences (Hartrick-Doane 2014).

## Review limitations

Although the authors were rigorous in the review process, by using a recognised methodology it is possible that some studies could have been missed. Publication in English as an inclusion criterion may have led to the omission of important interventional studies published in other languages. Most of the studies identified in this scoping review were conducted in the ICU, thus limiting translation to other acute care settings especially the ED, which is characterised by transient care and focuses on rapid throughput of patients.

## Conclusion

The findings of this review reiterate the fact that there is a scarcity of interventional studies focusing on genuine connection between families and HCPs in acute care settings. The interventions of the reviewed studies indicated variability regarding inclusion of the elements of relational practice and the nature of family engagement in the interventions. Taking into account the positive outcomes of family and HCP collaboration in the reviewed studies, it is recommended that ongoing training and education to capacitate HCPs relationally should be a major component in future interventions seeking to promote relational practice with families.
